# Circulating Calprotectin as a Predictive and Severity Biomarker in Patients with COVID-19

**DOI:** 10.3390/diagnostics12061324

**Published:** 2022-05-27

**Authors:** Gary L. Norman, Sherwin A. Navaz, Yogendra Kanthi, Roger Albesa, Michael Mahler, Jason S. Knight, Yu Zuo

**Affiliations:** 1 Headquarters & Technology Center Autoimmunity, Werfen, San Diego, CA 92131, USA; ralbesa@werfen.com (R.A.); mmahler@werfen.com (M.M.); 2 Division of Rheumatology, Department of Internal Medicine, University of Michigan, Ann Arbor, MI 48109, USA; sherwnav@med.umich.edu (S.A.N.); jsknight@med.umich.edu (J.S.K.); yzu@med.umich.edu (Y.Z.); 3 Division of Intramural Research, National Heart, Lung, and Blood Institute, Bethesda, MD 20892, USA; yogen.kanthi@nih.gov

**Keywords:** calprotectin, serum, biomarker, COVID-19, SARS-CoV-2, severity, predictive, chemiluminescent immunoassay, S100A8/S100A9

## Abstract

Background: New tools for the assessment and prediction of the severity of hospitalized COVID-19 patients can help direct limited resources to patients with the greatest need. Circulating levels of calprotectin (S100A8/S100A9) reflect inflammatory activity in multiple conditions, and have been described as being elevated in COVID-19 patients, but their measurement is not routinely utilized. The aim of our study was to assess the practical and predictive value of measuring circulating calprotectin levels in patients at admission and during their hospitalization. Methods: Circulating calprotectin levels were measured in 157 hospitalized patients with COVID-19 using an automated quantitative chemiluminescent assay. Results: Circulating calprotectin levels were strongly correlated with changing respiratory supplementation needs of patients. The overall trajectory of circulating calprotectin levels generally correlated with patient improvement or deterioration. Conclusions: Routine measurement of circulating calprotectin levels may offer a valuable tool to assess and monitor hospitalized patients with COVID-19, as well as other acute inflammatory conditions.

## 1. Introduction

Although increasing immunity levels due to past infection and vaccination have resulted in declining COVID-19 mortality in most regions, high infection rates continue, the severe acute respiratory syndrome coronavirus 2 (SARS-CoV-2) virus continues to evolve, and individuals remain at risk of progressing to severe disease. This is of particular relevance in light of emerging variants of concern (VOCs) such as Delta (B.1.617.2) or, most recently, the Omicron variant (B.1.1.529) [1]. Biomarkers that can help identify and stratify patients early in the disease course who are at risk of more severe disease, and which can assist in monitoring patient improvement or deterioration, would be of significant value for the clinical management of patients with COVID-19 by guiding resource allocation. Calprotectin, a heterodimer of the S100A8/S100A9 proteins, is abundantly expressed by neutrophils, monocytes, and platelets [2,3]. Levels of circulating calprotectin (cCP) increase as a result of inflammation, infection, and trauma [4]. Measurement of levels of fecal calprotectin (fCP) has rapidly become a routine test for assessing and monitoring mucosal inflammation in patients with inflammatory bowel disease. Increased levels of fCP have been investigated in patients with COVID-19, and are generally correlated with increased severity [5,6]. 

Several studies have shown an association between cCP and intensive care unit (ICU) admission, mechanical ventilation, and mortality, but there has been little examination of its potential to monitor improving or deteriorating patient status [7,8,9,10]. We therefore conducted a study to confirm previous observations and, importantly, to assess the trajectory of cCP levels with changing respiratory requirements in a series of patients with longitudinal blood specimens. 

## 2. Materials and Methods

Sera collected from 157 patients admitted to University of Michigan Hospital during spring 2020 with a diagnosis of COVID-19 confirmed by an FDA-approved RNA test were tested with a sensitive, quantitative, automated circulating calprotectin chemiluminescent immunoassay (QUANTA Flash^®^, Inova Diagnostics, San Diego, CA, USA; Investigational Use Only in USA, CE (Conformitè Europëenne) marked for in vitro diagnostics) on the BIO-FLASH^®^ instrument (Biokit SA, Barcelona, Spain). This assay utilizes paramagnetic beads coated with calprotectin-specific antibodies to capture calprotectin. Briefly, after incubation with the patient specimen, followed by washing steps, bound calprotectin is detected by an anti-calprotectin monoclonal antibody conjugated to isoluminol. Upon exposure to a triggering reagent, bound isoluminol generates a luminescent signal, which is detected by the instrument as relative light units (RLUs). The RLU values are proportional to the amount of calprotectin captured on the paramagnetic beads. Using a predefined, lot-specific master curve and the results of three calibrators, the instrument software calculates μg/mL for each sample. The analytic measuring range of the assay is 0.18 to 22.76 µg/mL. Samples with cCP over 22.76 µg/mL can be automatically diluted 10-fold and re-run by the instrument to allow values up to 227.60 µg/mL to be measured. According to the manufacturer, the precision of the cCP assay evaluated according to CLSI EP05-A3 was as follows: repeatability (CV 2.1–3.3%), inter-run (CV 1.3–2.6%), inter-day (CV 2.1–4.5%), and intra-laboratory precision (CV 3.1–5.1%).

All patients were classified by ventilation status at the time of the first specimen collection, as follows: room air (RA, *n* = 29), nasal cannula (NC, *n* = 37), high-flow oxygen (HFO, *n* = 12), and mechanical ventilation (MV, *n* = 79). RA, NC, HFO, and MV would be equivalent by the 10-point WHO classification scheme to scores of 4, 5, 6, and 7–9, respectively, and by the alternative 8-point system to scores of 3, 4, 5, and 6–7, respectively [11,12]. Longitudinal specimens (3–27 per patient) were available for 20 patients. For these patients, available results on D-dimer (normal reference range (NRR: < 0.59 mg/mL), fibrinogen (NNR: 150–450 mg/mL), C-reactive protein (CRP; NRR: 0.0–0.6 mg/mL), ferritin (NRR: 6.0–155.0 ng/mL), and lactate dehydrogenase (LDH; NRR: 120–240 IU/L) determined by Michigan Medicine Laboratories (University of Michigan) using FDA-cleared assays were collected. IL-6 was infrequently measured, and few results were available. Circulating calprotectin levels were also determined in 39 healthy individuals. All specimens were processed within 4–6 h of venipuncture, stored at 5 °C for up to 5 days, and frozen at −20 °C or below. In addition to data in the manufacturer’s direction insert, the stability of cCP measurements using this assay on samples kept at 2–8 °C for up to 7 days was recently confirmed [13]. This study was approved by the University of Michigan Institutional Review Board (HUM00179409). 

Analyse-it for Microsoft Excel (version 5.90, Leeds, UK) and GraphPad Prism (version 5.03, San Diego, CA, USA) were used for statistical analysis and graphical presentation. Wilcoxon, Mann–Whitney, and ANOVA tests were used to compare categorical variables; the Mann–Whitney test was used to analyze differences between groups. Receiver operating characteristic (ROC) analysis was conducted to assess the diagnostic performance of cCP (using DataLab, Werfen, Barcelona, Spain); *p*-values < 0.05 were considered statistically significant, and 95% confidence intervals were calculated.

## 3. Results

### 3.1. Characteristics of the Patient Cohort

The characteristics of the study population are summarized in Table 1. The ages of the 157 patients ranged from 16 to 90 years old. The mean age was 58 years old, 41% were female, and 39% were Black/African-American. As detailed in Table 1, heart disease, lung disease, and obesity were the most common comorbidities, with each found in 55% of the study population. Venous thrombosis and arterial thrombosis were found in 7% and 0% of the patients, respectively. Ultimately, 78% of the patients were discharged, while 22% died.

### 3.2. Circulating Calprotectin Levels Were Correlated with the Degree of Respiratory Supplementation and Progression to Mechanical Ventilation

**Figure 1:**
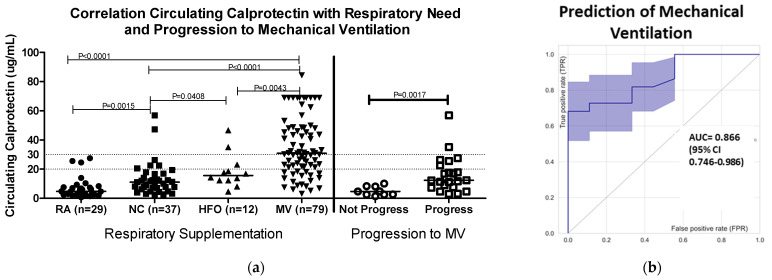
Circulating calprotectin levels (**a**) in relation to levels of oxygen supplementation (left-hand side of figure) and progression to mechanical ventilation (MV) at ≥ 1 day post-admission. Patients who progressed (N = 22) had significantly higher cCP levels compared to individuals (N = 9) who did not progress (Mann–Whitney, *p* = 0.0017). Dotted lines at 20 and 30 µg/mL indicate cutoffs referred for prediction of MV described in Section 3.1. (**b**) Receiver operating characteristic (ROC) curve analysis of cCP in relation to progression to MV. The area under the curve (AUC) was 0.866 (95% CI 0.746–0.986), showing strong discrimination between patients who progressed to MV and those who did not progress. Abbreviations: RA, room air; NC, nasal cannula; HFO, high-flow oxygen; MV, mechanical ventilation.

Patients with COVID-19 had increased median levels of cCP (4.8 µg/mL) compared to the manufacturer’s cutoff of 2 µg/mL for healthy individuals. In the present study, the median cCP level measured in 39 healthy controls was 1.2 µg/mL. Higher cCP levels were significantly associated with increased need for oxygen supplementation (median cCP levels: NC 11.13 µg/mL, HFO 15.62 µg/mL, and MV 30.87 µg/mL (ANOVA *p* < 0.0001)) (Figure 1a). ROC analysis showed strong discrimination (area under the curve, AUC = 0.85, 95% CI 0.79–0.91), and an odds ratio (OR) of 31.5 (1.6–618.5) between patients receiving MV vs. patients who did not (Figure 1b). At a cutoff of > 20 µg/mL, cCP had sensitivity of 65.3%, specificity of 89.3%, and OR of 15.7. A higher cutoff of > 30 µg/mL increased the specificity for MV to 96.6%, OR to 20.8, and positive predictive value (PPV) to 95.7%, albeit with a reduction in sensitivity to 43.6%. The mortality of patients with cCP levels > 20 µg/mL at admission was 27.7% (20/72), compared to 14.1% (12/85) for those with cCP < 20 µg/mL. 

The ability of cCP to predict progression to MV was assessed in 31 patients not on MV at baseline, and with ≥24 h between blood draw and the nadir oxygenation level recorded during hospitalization. The cCP values were significantly higher in patients who progressed compared to those who did not progress to require MV (median 12.35 vs. 4.74 µg/mL, Mann–Whitney *p* = 0.0017). ROC analysis showed an AUC of 0.866 (95% confidence interval 0.746–0.986) (Figure 1b). Patients with baseline cCP > 20 µg/mL were almost twice as likely to severely deteriorate during hospitalization compared to those with baseline < 20 µg/mL (OR 2.1, 95% CI 0.98–4.63). Furthermore, median levels of cCP were almost twice as high in the baseline specimens of those who died compared to those who recovered.

### 3.3. Circulating Calprotectin Levels Were Correlated with the Degree of Respiratory Supplementation and Progression to Mechanical Ventilation

**Figure 2:**
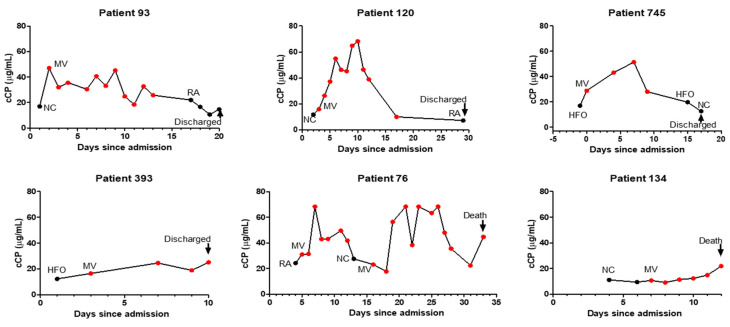
Profiles of longitudinal changes, corresponding respiratory supplementation, and final outcomes of representative patients with COVID-19. Abbreviations: RA, room air; NC, nasal cannula; HFO, high-flow oxygen; MV, mechanical ventilation.

Examining 20 patients with longitudinal measurements showed that increasing or declining cCP levels were generally correlated with respiratory deterioration or improvement, respectively (Figure 2). Circulating CP levels declined in 12/15 patients prior to discharge, while increasing cCP levels were observed in 3/5 patients who ultimately died. Interestingly, two patients with the 2nd and 3rd highest cCP levels among the 157 patients did not progress during their hospitalization. Their outcomes following discharge are unknown. 

### 3.4. Profile of Circulating Calprotectin and Additional Biomarker Results Are Intriguing, but Significance Needs Further Study

**Figure 3:**
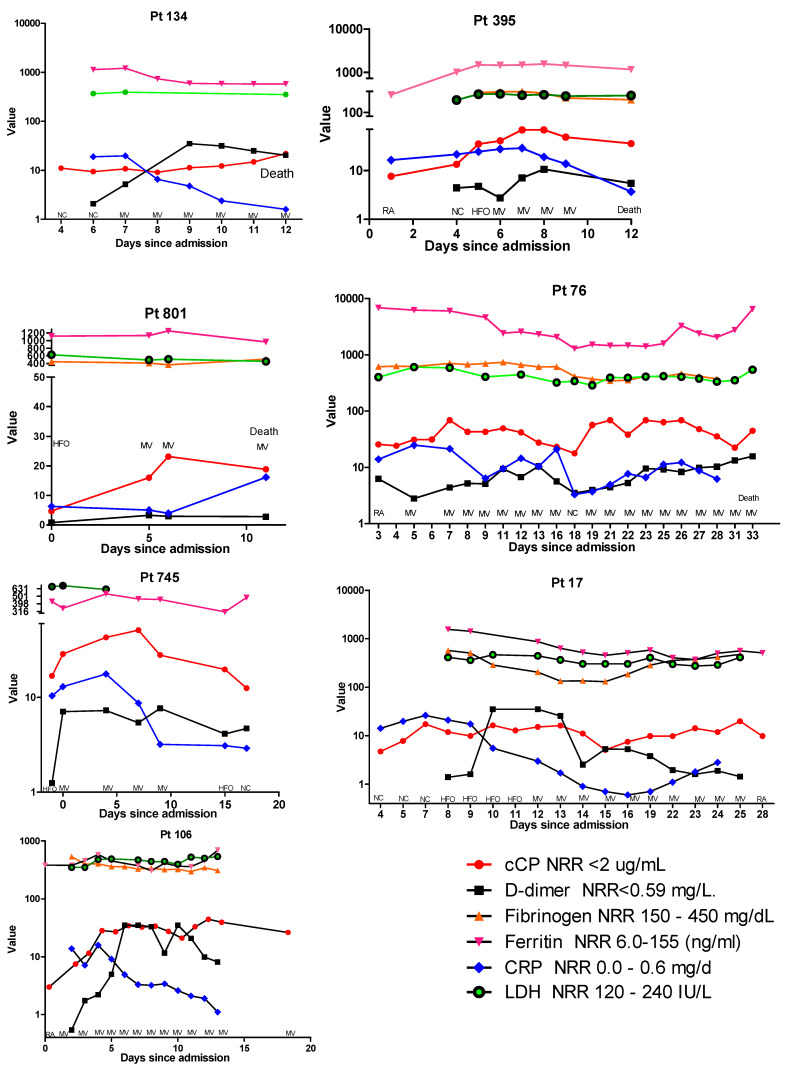
Comparative profiles of longitudinal biomarker changes, corresponding respiratory supplementation, and final outcomes of 4 hospitalized patients with COVID-19 who died (patients 134, 395, 801, and 76) and 3 patients who recovered (patients 745, 17, and 106). As a result of the widely different ranges of the various assays, the y-axis is presented in logarithmic scale, and in some panels has been split to accommodate very high values. Abbreviations: RA, room air; NC, nasal cannula; HFO, high-flow oxygen; MV, mechanical ventilation; NRR, normal reference range; CRP, C-reactive protein; LDH, lactate dehydrogenase.

In addition to cCP, results for D-dimer, fibrinogen, CRP, ferritin, and LDH were available for a small subset of the longitudinally followed patients. Examination of these patients revealed a number or different profiles. In Figure 2, the cCP levels in patient 134 show a gradual increase until their death at 12 days post-admission. When results for the other biomarkers are added to this profile, as shown in Figure 3, one can see that D-dimer increased until day 8, at which time it appeared to begin declining. At day 8 ferritin and LDH remained elevated but stable. In contrast, CRP was highest at day 6, and declined from 19.7 to 1.6 μg/mL prior to death. A similar trend was observed in patient 395, where at day 6 CRP began to decline, while cCP remained high until death. In contrast, patient 801 showed both cCP and CRP values rising from admission to death. Examination of the biomarker profiles of patients who were ultimately discharged also showed varied patterns. Patient 745 showed a very clear rise and fall in cCP with changing respiratory need. CRP also rose modestly and fell until discharge. In patient 17, cCP values were high at admission, increased modestly with increasing respiratory need, fell at day 15, and then appeared to slowly increase until day 25. CRP showed a similar initial rise during the first week, and then progressively declined until day 15 when, like cCP, it began to rise. D-dimer peaked on days 10–13, declined on day 14, rose on day 15, and then declined until discharge at day 28. Patient 106 showed a progressive decline in CRP from 4 days post-admission, but an opposite rise in both D-dimer and cCP. While D-dimer appeared to start declining at day 11, cCP remained high. 

## 4. Discussion

Despite progress in understanding the pathogenesis of COVID-19, the development of vaccines, and increasing experience in managing patients, COVID-19 remains a critical healthcare problem worldwide. While intense efforts to understand and predict the differential impact of SARS-CoV-2 infection on individuals have resulted in an expanding trove of data on gene, protein, and cellular processes in patients with COVID-19, front-line care of patients still focuses on the use of practical, well-understood, and easily automated routine laboratory tests [8,14,15,16]. The strong inflammatory component of COVID-19 has led to interest in measuring cytokines such as IL-6, as well as other differentially expressed biomarkers, but these assays are not routinely available in clinical laboratories [17].

Interest in calprotectin as a biomarker of inflammation has surged with its increasing availability on ELISA and automated platforms, and with better understanding of the important pre-analytical requirements for reproducible measurement [13,18,19,20]. Recognition that changes in calprotectin levels can indicate increasing or decreasing inflammatory changes in diverse inflammatory conditions has led to growing interest in cCP as a practical and clinically useful biomarker for patient management, and as a potential therapeutic target [4,20,21,22,23,24]. However, despite multiple recent studies showing that cCP levels correlate better with the severity of COVID-19 than a host of other biomarkers—including IL-6 and CRP—cCP is seldom used to assess and follow COVID-19 patients, likely due to unfamiliarity and, until recently, the unavailability of automated, quantitative practical assays [7,8,9,23,24,25]. The importance of our study is that it provides support for expanding assessment and consideration of the potential value of cCP measurement as a routine assay at hospital admission for risk stratification and monitoring of patients. While previous studies have generally shown differences between patients grouped by severity (i.e., healthy, mild, severe, fatal), we believe ours is the first to longitudinally follow and present profiles of the changes in respiratory requirements and cCP levels of individual patients during their hospitalization (Figure 2). While at admission many patients may appear to be at a similar risk of progression, our results demonstrate that patients with high levels of cCP at admission are at higher risk of progression and poor outcomes, consistent with the findings of previous studies. High levels of cCP in patients not on MV at admission were predictive of the potential need for MV. Following submission of this manuscript, a new study has demonstrated the association of increased cCP levels with COVID-19 severity and the serial increase in cCP levels from baseline in patients who progressed to more severe disease, similar to the results we present here [26]. Figure 2 shows several patients with high levels of cCP at admission and initially on RA, NC, or HFO, who subsequently progressed to MV. High levels of cCP can therefore alert clinicians to patients who may potentially require more aggressive monitoring and management. Figure 2 graphically illustrates the potential value of monitoring cCP during hospitalization as an indicator of patient improvement or deterioration. Our data clearly demonstrate by multiple measures—including required and predicted respiratory support, as well as survival—that high or increasing levels of cCP are associated with more severe disease. The increasing levels of calprotectin seen in several patients (Figure 2) prior to death fit with the observation of high expression levels of calprotectin in postmortem lung tissue from deceased COVID-19 patients in two recent studies [15,27]. In one of these studies, it was noted that the deceased patients had a low viral load, suggesting that death was not a result of the viral infection but, rather, of uncontrolled hyperinflammatory processes—presumably reflected in the high calprotectin levels [15]. The recent study by Kassianidis speculated that the improved outcomes in COVID-19 patients treated with dexamethasone may be at least partially a result of its effect in decreasing neutrophils’ production of calprotectin [26,28]. The utilization of drugs to reduce cCP levels may prove to be a useful area of future development. Despite the strong correlation of cCP levels with severe disease and poor outcomes, the two patients in our study discharged with very high cCP levels highlight that other factors beyond hematopoietic cell-mediated inflammation may impact progression in some patients. It is tempting to speculate that the patients discharged with persistently high calprotectin levels—such as patient 106 in Figure 2—may be patients who will develop “long COVID-19”.

The trajectory of cCP levels generally appears to be correlated with patient outcome. Moreover, monitoring changes may provide actionable information to predict patient improvement or deterioration, and may help to guide clinical management. While a recent publication questioned the incremental benefit of cCP as a predictor of severe COVID-19 compared to CRP, the study used a different cCP assay, and focused on a review of 3280 ambulatory patients where only 6.8% had a final diagnosis of COVID-19 [29]. In this situation, CRP and other conventional biomarkers, along with clinical assessment, may be adequate. However, in patients with serious illness and those admitted to hospital care, measurement of cCP may aid in patient stratification, monitoring, and resource allocation. We compared the results of CRP, D-dimer, ferritin, fibrinogen, and LDH, in addition to cCP, in a small group of longitudinally followed patients (Figure 3). While a number of patterns were apparent, as described in the Results section, we had too few patients to reach conclusions. A larger series of patients may clarify significant profiles and their prognostic value. Although a limitation of this study is that the cohort was collected early in the pandemic, the conclusion that higher cCP levels represent an early biomarker of severe COVID-19 and an increased risk of progression remains strong. While the treatment and management of COVID-19 patients has progressed since the beginning of the pandemic, larger studies on recently collected cohorts are now needed to confirm the continued significance of our observations, and to both establish and refine the value of cCP for routine management of hospitalized patients with COVID-19, its value when combined with other biomarkers, and its value for patients with other acute inflammatory conditions.

## Figures and Tables

**Table 1:** Demographic and clinical characteristics of hospitalized COVID-19 patients.

Demographics		
Number	157	
Age (years) *	58 ± 17	(16–90)
Female	65	(41%)
White/Caucasian	70	(45%)
Black/African-American	62	(39%)
**Comorbidities**		
Diabetes	61	(39%)
Heart disease	87	(55%)
Renal disease	67	(43%)
Lung disease	87	(55%)
Autoimmune	4	(3%)
Cancer	20	(13%)
History of stroke	6	(4%)
Obesity	86	(55%)
Hypertension	77	(49%)
History of smoking	37	(24%)
**In-hospital thrombosis**		
Arterial thrombosis	0	
Venous thrombosis	11	(7%)
**Final outcome**		
Discharged	123	(78%)
Death	34	(22%)

* Mean ± standard deviation (range).
